# Personalized Orbital Fracture Repair: Enhancing 3D-Printed Titanium Mesh with Biomimetic 3D-Printed HAP-COL Frameworks

**DOI:** 10.22336/rjo.2026.33

**Published:** 2026

**Authors:** Ovidiu Lazăr, Sînziana Istrate, Alina Popa-Cherecheanu, Laura-Mădălina Cursaru, Diana Popescu, Ana Maria Catrina, Cerasela Hăidoiu, Vladimir Suhăianu, Seclaman Edward, Istetia Rami

**Affiliations:** 1 “Carol Davila” University of Medicine and Pharmacy, Bucharest, Romania; 2 Department of Anesthesia and Critical Care, Life Memorial Hospital, Bucharest, Romania; 3 Bine Clinic, Bucharest, Romania; 4 Department of Ophthalmology, Emergency University Hospital, Bucharest, Romania; 5 Nanostructured Materials Laboratory, National R&D Institute for Nonferrous and Rare Metals, Pantelimon, Romania; 6 “Cantacuzino” National Military Medical Institute for Research and Development, Bucharest, Romania; 7 Department of Biochemistry, “Victor Babeș” University of Medicine and Pharmacy Timișoara, Timişoara, Romania

**Keywords:** orbital wall reconstruction, hydroxyapatite-collagen composite, 3D-printed titanium implant, robocasting, biocompatibility, AM = additive manufacturing, CAD = computer-aided design, CAD-CAM = computer-aided design and manufacturing, DIW = direct ink writing, EDS = energy-dispersive spectroscopy, FAAS = flame atomic absorption spectrometry, FBS = fetal bovine serum, FT-IR = Fourier-transform infrared spectroscopy, HAp = hydroxyapatite, HAP-COL = hydroxyapatite-collagen composite, hMSC = human mesenchymal stem cell, ICP-OES = inductively coupled plasma optical emission spectrometry, MTT = 3-(4, 5-dimethyl-2-thiazolyl)-2, 5-diphenyl-2H-tetrazolium bromide, NHOst = normal human osteoblasts, OD = optical density, SEM = scanning electron microscopy, XRD = X-ray diffraction

## Abstract

**Objective:**

Orbital wall fractures require precise reconstruction to restore orbital volume and visual function, and bio-inert titanium implants benefit substantially from osteoconductive surface modifications. This study evaluated the biocompatibility and osteogenic potential of hydroxyapatite-collagen (HAP-COL) composites used in titanium implants intended for orbital fracture repair, comparing a sprayed HAP coating with a robocasting-deposited, 3D-printed HAP-COL layer.

**Methods:**

HAp-COL hybrid powder (HAp:COL mass ratio 4:1) was prepared by hydrothermal synthesis at 100 °C and 10^4^ Pa, followed by spray drying, and characterized by FAAS, ICP-OES, FT-IR, XRD, and SEM-EDS. Two implant configurations were tested: Sample 1 (titanium with sprayed HAp) and Sample 2 (titanium with three robocasting-deposited 3D-printed HAP-COL layers). Cytotoxicity was assessed on a normal human osteoblast cell line (NHOst, Lonza CC-2538) in accordance with ISO 10993-12, using sample extracts at stock concentration and binary dilutions (1/2, 1/4, 1/8) and quantified by MTT assay at 24 and 48 hours.

**Results:**

Both samples showed dose- and time-dependent viability profiles within the acceptable limits of ISO 10993-5. Sample 1 viability ranged from 70.62% (stock) to 89.72% (1/8) at 24 h, and from 77.28% to 93.63% at 48 h. Sample 2 consistently outperformed Sample 1, with values from 80.61% to 91.28% at 24 h and from 83.35% to 96.47% at 48 h.

**Discussion:**

Both HAP-COL application techniques proved highly biocompatible and compliant with ISO 10993-5 non-cytotoxic safety limits. However, the 3D-printed robocast layer (Sample 2) consistently outperformed the sprayed coating, exhibiting superior osteoblast viability and proliferation. This demonstrates that a layered, biomimetic robocast architecture optimizes cell survival and successfully mitigates the bio-inert properties of standard titanium.

**Conclusions:**

The 3D-printed HAP-COL coating produced by robocasting exhibited superior biocompatibility and osteoconductive behavior compared with the sprayed alternative, supporting sustained osteoblast proliferation. HAP-COL robocast layers on titanium represent promising candidates for personalized orbital wall reconstruction, although in vivo validation remains necessary.

## Introduction

Orbital fractures present a significant surgical challenge, requiring precise reconstruction of bone walls to restore orbital volume and visual function, aspects that can be optimized using custom titanium mesh implants coated with hybrid composites. These hybrid structures, often synthesized via high-pressure hydrothermal techniques, exhibit high biocompatibility, supporting fibroblast proliferation and mesenchymal stem cell growth [1]. At the same time, the incorporation of Fe and Mn ions into these mineral structures has demonstrated the potential to enhance osteoinductive properties, thereby facilitating superior tissue integration at the implant-host bone interface [2]. In this context, bioprinting technologies enable the replication of the complex architecture of bone tissue using advanced bio-inks that provide robust mechanical support and facilitate the progressive maturation of cell constructs. However, integrating these materials requires rigorous attention to interactions among components, as adhesion between dissimilar materials is a determining factor in the mechanical integrity of the resulting composites [3].

Bone tissue engineering presents a promising avenue for regenerative medicine, particularly in addressing complex skeletal defects that necessitate the integration of biological and mechanical properties [4]. Central to this approach is the development of biomaterials that can mimic the native bone extracellular matrix, thereby promoting cell viability, proliferation, and differentiation towards an osteogenic lineage [5]. Hydroxyapatite and collagen compounds are particularly attractive due to their inherent osteoconductive and osteoinductive properties, closely mirroring the inorganic and organic phases of natural bone, respectively [6]. The exceptional biocompatibility, osteoconductivity, and potential osteoinductivity of bioceramics such as hydroxyapatite significantly enhance their ability to promote bone regeneration by modulating in vivo conditions [7]. This is especially crucial given that widely used materials, such as titanium alloys, despite their favorable mechanical properties, are bio-inert and require modifications to stimulate effective bone regeneration [8,9]. Consequently, surface modifications, including the application of hydroxyapatite coatings, have emerged as a pivotal strategy to enhance the osteointegration and overall biological performance of titanium-based implants [10]. For instance, the application of hydroxyapatite, in conjunction with bovine collagen, as a coating significantly enhances the bioactivity of titanium substrates, promoting apatite growth and improving mechanical properties, albeit with a reduction in compressive strength due to collagen incorporation [11]. However, the precise architectural design of these composite materials, particularly in 3D-printed scaffolds, is crucial for optimizing both mechanical integrity and biological function, as the framework can influence cellular differentiation [12,13]. This article, therefore, aims to provide an exhaustive overview of the fabrication, characterization, and biological evaluation of hydroxyapatite-collagen composites, ranging from powder extracts to advanced 3D-printed titanium implants, with a focus on their osteogenic potential and biocompatibility.

### State of the art

Orbital fractures currently benefit from titanium-based solutions for their mechanical strength, but integrating nanostructured hydroxyapatite coatings is imperative to enhance osteoconductivity and optimize clinical outcomes [14]. This technological approach leverages the biomimetic properties of hydroxyapatite, which facilitate the formation of direct chemical bonds with native bone tissue and support the cellular adhesion necessary for effective repair [15,16]. In addition, the use of collagen and hydroxyapatite-based compositions in tissue engineering is a promising step forward.

Current research highlights the development of biocomposites that address the inherent brittleness of pure hydroxyapatite by integrating natural or synthetic polymers to achieve a scaffold architecture that closely mimics the complex, porous nature of native trabecular bone [17,18]. These advancements leverage nanotechnology-based frameworks to functionalize proteins and growth factors, which are essential for stimulating osteogenic cell adhesion and differentiation [19]. Furthermore, incorporating these composite materials into titanium-based systems aims to bridge the gap between mechanical load-bearing capacity and the biochemical cues required for long-term osseointegration [20,21]. Such hybrid systems often capitalize on the nano-regime properties of hydroxyapatite, which significantly improve cell adherence and subsequent proliferation compared to micro-scale alternatives [22]. Moreover, advanced manufacturing techniques, such as 3D bioprinting, enable the creation of highly porous, interconnected architectures that facilitate critical nutrient exchange and cell infiltration, addressing historical challenges in tissue integration [23]. Despite these advancements, ensuring uniform deposition of mineral phases within intricate 3D-printed geometries remains a significant challenge, often leading to heterogeneous osteoconductivity [24]. To mitigate these limitations, recent studies have shifted toward indirect additive manufacturing or controlled cross-linking strategies, such as the use of genipin, to stabilize collagen-hydroxyapatite matrices without compromising their structural integrity [25]. Furthermore, the nature-inspired biomineralization approach facilitates the precipitation of nano-hydroxyapatite crystals directly within the collagen fiber network, effectively mimicking the hierarchical assembly of natural bone tissue [26].

### 3D printing technique

Selecting a suitable methodology, such as extrusion or stereolithography, is essential to balance mechanical properties with the required biocompatibility, given that composite inks based on natural and inorganic polymers have become a focal point of current research [27].

**Fig. 1:**
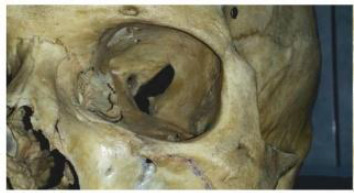
Anatomy of the orbit; the thickness of the orbital walls is visible

The orbit is a highly fragile region (Fig. 1) where the complex geometry and limited space require materials with a high capacity to retain their post-extrusion shape to prevent structural collapse of the scaffold [28,29]. In this sense, the shear-thinning behavior becomes an essential rheological attribute, allowing viscosity to decrease under pressure during extrusion, followed by rapid recovery to stabilize the deposited geometry [30]. In addition, adapting the paste composition by optimizing the solid-phase loading and polymer fractions ensures superior dimensional stability, which is essential to avoid post-printing deformations under the influence of gravity [31,32]. Thus, the growing trend toward adopting additive manufacturing at the expense of traditional methods is driven by several important advantages, including the ability to achieve complex geometries with high precision, material savings, flexibility, and design customization. However, the limited availability of materials is a major disadvantage of the method, which is why researchers have, in recent years, turned their attention to developing new materials suitable for additive manufacturing processes. These advanced materials aim to improve mechanical properties and in vivo degradation rates, enabling the creation of implants that support tissue regeneration in a biomimetic way [33]. In fact, the integration of computer-aided design strategies enables precise control over the internal architecture and pore distribution, critical aspects for optimizing the interaction between the implant and the host biological environment [34,35].

Titanium plates used for orbital fractures often have limitations in anatomical adaptability and the risk of soft-tissue entrapment, which has led to a transition to custom 3D-printed implants to ensure optimal geometric fit and superior primary stability [36,37]. This transition to customized solutions is supported by additive manufacturing technologies' ability to reduce the need for complex bone grafting interventions, thereby minimizing the risks associated with secondary defects [38]. In addition, the integration of CAD-CAM technologies enables the design of mesh structures with anatomical precision, facilitating extensive bone regeneration in complex craniofacial areas [39].

Robocasting, or direct ink writing (DIW), is an additive manufacturing technique in which a paste-form material is extruded layer by layer through a nozzle to produce the previously designed structure in CAD software (e.g., SolidWorks 2019). Among the advantages of using this technique are the lower cost of devices and raw materials, and the adaptability and variability of the materials that can be printed, compared with other AM technologies, which often require narrow particle-size distributions, excellent fluidity, and specific light absorption. In addition, the versatility of this approach enables the use of complex hydrogel-ceramic composite-based inks that support high cell viability during deposition while facilitating the creation of self-healing architectures with improved mechanical properties [40]. At the same time, precise control of rheological parameters, such as apparent viscosity and flow threshold, remains fundamental to guarantee geometric fidelity and shape retention during layered deposition [41]. Also, optimizing these rheological parameters is critical to preventing nozzle clogging, which frequently occurs when bio-inks contain high cell densities or large particle sizes [42]. In this respect, the precise adjustment of the shear forces applied during extrusion ensures the die’s structural integrity.

**Fig. 2:**
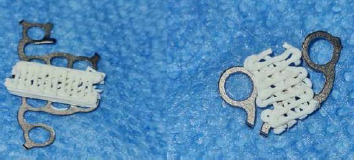
3D-printed HAP-COL structures placed on the titanium plate by the robocasting technique

Moreover, unlike other additive manufacturing (AM) methods, robocasting can produce 3D-printed structures at room temperature without requiring lasers or ultraviolet light. The quality of the deposition paste depends solely on its rheological properties, which can be controlled during preparation (Fig. 2). This technique also facilitates the processing of high-solid-load suspensions, which is essential to ensure the density and mechanical performance required for bone regeneration applications in areas subject to structural loads [43,44].

## Materials and methods

### Hydrothermal synthesis of hydroxyapatite-collagen (HAP-COL) hybrid powder

Hydroxyapatite-collagen powder was obtained by hydrothermal synthesis from calcium nitrate, ammonium dihydrogen phosphate, and hydrolyzed collagen, all dissolved in distilled water and magnetically stirred. 25% ammonia was gradually added to the solution thus obtained until an alkaline pH (pH = 10) was reached. The resulting suspension was transferred to the Teflon vessel of the Berghof autoclave for hydrothermal synthesis under controlled temperature and pressure conditions (100 °C and 10^4^ Pa). The obtained precipitate was separated from the aqueous phase of the suspension by vacuum filtration and washing until pH = 7, followed by spray drying using the Lab PLANT equipment. A white, granulated powder based on hydroxyapatite and collagen (HAp:COL mass ratio = 4:1) was obtained.

### Physicochemical, structural, and morphological characterization of the hybrid powder

The following physicochemical and morphological analysis methods characterized the hybrid powder obtained as described above:

- *Quantitative chemical analysis*. The determination of the Ca content was performed by the flame atomic absorption spectrometry (FAAS) method, and the P content by the inductively coupled plasma optical emission spectrometry method (ICP-OES).
- *FT-IR spectroscopy analysis*. Fourier identified the presence of functional groups (chemical structure of the powders) using transform infrared spectroscopy (FT-IR) with an ABB MB 3000 FT-IR spectrometer (Canada) equipped with an EasiDiff device for powders. The solid sample is mixed with KBr to achieve a 1% (by mass) concentration. The mixture thus obtained is ground for 10-15 minutes to obtain fine, homogeneous particles. For data acquisition, 64 scans were performed at a resolution of 4 cm^−1^, over the range 550-4000 cm^−1^ in transmittance mode. Experimental data were processed using the Horizon MB™ FTIR software.
- *X-ray diffraction analysis (XRD)*. XRD was used to analyze the obtained powder to determine the crystalline phases. For the measurements, a Bruker-AXS D8 ADVANCE Bragg-Brentano diffractometer with radiation source (Cu) and SOL X detector in vertical geometry θ-θ, equipped with BRUKER AXS software, was used. The diffraction spectra were acquired over the angular range 6-70° in continuous mode with a step size of 0.02°. The data processing was performed using DIFFRAC.EVA VER.5 2019 program from the DIFFRAC.SUITE.EVA software package and the ICDD PDF-5+2024 database.
- *Electron microscopy (SEM) analysis*. The study of particle morphology was carried out with an FEI Quanta 250 electron microscope in High Vacuum (HV) (ESEM) mode, using the secondary electron detector (ETD), the backscattered electron detector (CBS), and the energy-dispersive X-ray spectroscopy (EDS) detector.

Using the robocasting technique, 3 3D-printed layers of HAP-COL paste were deposited onto the titanium plates, thereby ensuring an optimized osteoconductive interface for the biological integration of the implant. Subsequently, the microstructural and compositional characterization of these coated scaffolds was performed by scanning electron microscopy and energy-dispersive spectroscopy, confirming the uniformity of the layer deposited on the metal surface [45].

### Cell line

To perform the cytotoxicity test on the 2 titanium implants with HAP-COL, we used a normal human osteoblast cell line (NHOst; Lonza code CC-2538). These cells were cultured in a specific growth medium supplemented with differentiating agents to assess the biocompatibility and osteoinductive potential of the composite scaffolds [46,47].

The osteoblast cell line was cultured in 25 cm^2^ cell culture vessels using osteoblast-specific growth medium, supplemented with 10% fetal bovine serum (FBS) and 50 μg/ml of gentamicin. Incubation conditions were maintained at 37°C in a 5% CO2-humidified atmosphere, with periodic monitoring of cell confluence to ensure viability before seeding onto titanium scaffolds. Subsequently, to validate the safety profile of the materials, cytotoxicity testing was conducted in accordance with international standards, confirming the coating’s biocompatibility in the cell line used.

The cells from 2 flasks of 25 cm^2^ were trypsinized with 0.025% Trypsin-EDTA, centrifuged, and the pellet resuspended in 5 ml of growth medium completely specific to the cell line. Initial cell counting was performed by staining with Trypan Blue 1:1 (50 μl cell suspension + 50 μl Trypan Blue). The cell suspension of osteoblasts had a concentration of 1.1 × 10^6^ cells/ml. Subsequently, the suspension was properly diluted to achieve the cell density required for seeding onto titanium samples, thereby ensuring optimal conditions for proliferation and osteogenic differentiation assays [48].

### Preparation of experimental models to be tested

Two experimental models were received for testing, namely:

- Sample 1 - titanium implant with sprayed HAP-COL;
- Sample 2 - titanium implant with 3D-printed HAP-COL.

The 2 test samples were placed in a 12-well cell culture plate containing a growth medium specific to the osteoblast cell line and incubated for 24 hours at 37°C in a shaking incubator. The weight-to-volume ratio was 200 mg/ml, in accordance with ISO 10993-12. After incubation, the stock solution was diluted in binary dilutions (1/2, 1/4, and 1/8). Subsequently, these extracts were used to expose osteoblast cultures to varying concentrations of the material, thereby enabling evaluation of cell viability using colorimetric assays [49].

### Method

For the cytotoxicity test, the cell suspension was cultured in 2 microplates with 96 flat-bottomed wells, 200 μl/well (2.2 × 10^5^ cells/well), and incubated in the CO^2^ incubator (5%) at 37°C for 24 hours. After this interval expired, the culture medium was replaced with extracts from titanium samples, and the cells were further incubated to assess cytotoxicity using specific colorimetric methods, such as Alamar Blue or PrestoBlue [50,51].

The next day, the medium was changed as follows:

- only 200 μl of complete growth medium was added to the blank wells;
- in the cell-control wells, the medium was removed, and 200 μl of complete, fresh growth medium was added;
- in the wells with the test sample, the medium was removed, and 200 μl of fresh medium containing different sample concentrations (200 mg/ml, 100 mg/ml, 50 mg/ml, and 25 mg/ml) was added.

Two culture microplates with 96 wells were prepared: one plate was incubated for 24 hours, and the other for 48 hours at 37°C and 5% CO^2^. Blank, cell control, and test samples were distributed (with each dilution) in duplicate (Table 1).

**Table 1:** Distribution of test samples on the microplate (each dilution in duplicate).

	1	2	3	4	5	6	7	8	9	10
A	Blank	NHOst control	Sample 1 stock	Sample 1 stock	Sample 1 1/2	Sample 1 1/2	Sample 1 1/4	Sample 1 1/4	Sample 1 1/8	Sample 1 1/8
B	Blank	NHOst control	Sample 2 stock	Sample 2 stock	Sample 2 1/2	Sample 2 1/2	Sample 2 1/4	Sample 2 1/4	Sample 2 1/8	Sample 2 1/8

After 24 and 48 hours, the 3-(4,5-dimethyl-2-thiazolyl)-2,5-diphenyl-2H-tetrazolium bromide (MTT) test was performed, which measures the conversion of MTT into a colored product in living cells. For this test, we used the MTT-based cell growth determination kit (Sigma), containing the MTT solution (5 mg/ml MTT in RPMI-1640 without phenol red) and the MTT solvent (0.1 N HCl in anhydrous isopropanol). Specifically, after the final incubation, the medium was replaced with 1 mg/ml MTT solution and left to react for 4 hours at 37°C, followed by the solubilization of the formed formazan crystals with DMSO [52,53]. The absorbance of the resulting solution was measured at 570 nm using a microplate reader, thereby quantifying the optical density, which is proportional to the number of viable cells [54,55].

The microplate was removed from the incubator, 20 μl of MTT/well (10% of the medium volume) was added, and it was incubated for 4 hours at 37°C in the dark with CO^2^. After 4 hours, the microplate was removed from the incubator, the medium was removed, and the MTT solvent (200 μl/well) was added. The optical density was read at 570 nm within 1 hour of adding the solvent using the EnSight™ Multimode Microplate Reader (PerkinElmer). The obtained data, expressed as percentages of cell viability relative to the negative control, were corrected by subtracting the absorbance of the blank wells [56].

## Results

For the interpretation of the results, the following formula was used:

% cell viability = [(OD positive control - OD blank) / (OD negative control - OD blank)] × 100

Where:

- positive control = cells + compound + MTT + MTT solvent;
- negative control = cells + MTT + MTT solvent;
- blank = (complete growth) medium + MTT + MTT solvent.

The negative control (cell control) and the samples (positive control) were run in 2 wells per compound concentration, and the arithmetic mean of the optical density readings at 570 nm was calculated.

The results are presented in Table 2.

**Table 2:** Cell viability results (MTT assay) for the two tested samples at 24 and 48 hours

Test sample	Viability (%)				Reading interval
	Stock	Dilution 1/2	Dilution 1/4	Dilution 1/8	
Sample 1	70.62	77.57	83.35	89.72	24 hours
Sample 2	80.61	82.86	87.95	91.28	
Sample 1	77.28	81.78	87.07	93.63	48 hours
Sample 2	83.35	85.01	88.44	96.47	

## Discussion

The results of cell viability in the case of the use of osteoblasts showed the following:

- The cytotoxicity of the tested samples was dose-dependent, with an increase in cell viability as the concentration of the extracts decreased, reaching maximum values at 1/8 dilution. At the same time, both samples exhibited high biocompatibility, with cell viability values remaining within acceptable limits per ISO 10993-5 guidelines, confirming the potential of these materials for clinical applications [57].
- Cell viability is lowest in culture wells with a concentration of 200 mg/ml of extract. It increases in direct proportion to the dilution of the test samples, demonstrating that the tested materials do not induce significant metabolic stress that compromises the integrity of cell populations at low concentrations [58]. Also, the differences observed between the 24- and 48-hour intervals suggest a progressive adaptation of NHOst cells to titanium extracts, reflecting a favorable biocompatibility profile in the medium term [59,60]. At the same time, the values obtained indicate a mild cytotoxicity, classified according to the standards as non-cytotoxic at high dilutions [61].
- Regarding the time of action of the compound on osteoblasts, an increase in cell viability is observed with the increase in the time of action, which indicates a sustained cell proliferation rate in the presence of the extracts during the 48 hours of monitoring. These data suggest that the analyzed materials support mitochondrial metabolic activity in osteoblasts without triggering inhibitory cytotoxic effects at the evaluated concentrations [62,63].
- Making a comparison between the 2 samples tested, it is observed that Sample 2 (titanium implant with 3D-printed HAP-COL) has a higher viability at both 24 hours and 48 hours. This superior trend in Sample 2 suggests improved biological compatibility and high osteoconductive potential, as evidenced by more efficient cell proliferation in the culture medium.

These results are consistent with studies confirming that titanium-based materials and related alloys promote osteoblast proliferation and exhibit excellent biocompatibility in vitro [64]. Although MTT assays provide valuable indicators of metabolic activity, there is a need to extend the assessments through complementary analyses, such as fluorescence microscopy, to visualize cell morphology and adhesion on implantable surfaces [65,66].

### Limitations and Future Directions

The authors note that while these *in vitro* results are promising, they must be interpreted with caution:

- In vivo translation: Laboratory cultures cannot account for local immune responses or systemic repair mechanisms. Therefore, future studies should integrate co-culture models or patient-derived primary cells to more accurately simulate the complex cellular environment encountered in clinical applications [67]. In addition, the long-term investigation of the mechanical properties and surface stability of implants, including resistance to bacterial adhesion, remains essential for validating the clinical success of these treatments [68]. Also, an analysis of vascularization dynamics and immunomodulatory potential would provide a more comprehensive insight into how these materials interact with the dynamic bone microenvironment [69].
- Cell source: Commercial primary cells may behave differently from patient-derived cells and can change over successive passages. Thus, validating the performance of these materials in more complex biological systems, such as animal models, is imperative to overcome the intrinsic limitations of cellular metabolism tests and to assess their potential for tissue integration accurately [70]. In this context, future studies should explore the influence of physiological loading and cell migration in native tissue using *ex vivo* models, thus overcoming the limits of static cell-only models [71]. These approaches align with international standards such as ISO 10993, which emphasize the need to evaluate medical devices using experimental models that reproduce complex biological interactions and mechanical loads specific to the *in vivo* environment [72]. In addition, given the potential interference between porous materials and reagents used in cytotoxicity assays, future research should employ tailored protocols to ensure accurate quantification and a clearer distinction between transient chemical effects and material-induced cytotoxicity.

## Conclusion

This study successfully demonstrated the synthesis of a hydroxyapatite-collagen (HAP-COL) hybrid powder via controlled hydrothermal processing and its application as an osteoconductive surface modification for titanium implants. The in vitro biological evaluations of a normal human osteoblast cell line established that both the sprayed coating and the 3D-printed robocast layer met the non-cytotoxic safety thresholds defined by ISO 10993-5. Both implant configurations elicited a favorable, dose-dependent cellular response, with the highest cell viability observed at the lowest extract concentrations.

Our study presents a step forward by validating a customized, robocast hydroxyapatite-collagen (HAP-COL) matrix that directly mimics the native bone extracellular matrix. By delivering an engineered surface that significantly enhances osteoblast proliferation compared with traditional spraying techniques, this approach offers a highly predictable pathway to restoring critical orbital dimensions. Ultimately, this technological integration paves the way for truly personalized oculoplastic solutions that can accelerate structural bone healing, reduce long-term implant migration, and ultimately optimize functional visual outcomes for patients suffering from complex midfacial trauma.

## References

[1] Vasile VA, Istrate S, Cursaru LM, Piticescu RM, Ghita AM, Popescu DM, Garhöfer G, Catrina AM, Spandole-Dinu S, Haidoiu C, Suhaianu V, Voinea OC, Dragut DV, Popa-Cherecheanu A (2024). A New Approach for Orbital Wall Reconstruction in a Rabbit Animal Model Using a Hybrid Hydroxyapatite-Collagen-Based Implant. Int J Mol Sci.

[2] Hong MH, Lee JH, Jung HS, Shin H, Shin H (2022). Biomineralization of bone tissue: calcium phosphate-based inorganics in collagen fibrillar organic matrices. Biomater Res.

[3] Koushik TM, Miller CM, Antunes E (2023). Bone Tissue Engineering Scaffolds: Function of Multi-Material Hierarchically Structured Scaffolds. Adv Healthc Mater.

[4] Martinez JS, Peterson S, Hoel CA, Erno DJ, Murray T, Boyd L, Her JH, Mclean N, Davis R, Ginty F, Duclos SJ, Davis BM, Parthasarathy G (2022). High resolution DLP stereolithography to fabricate biocompatible hydroxyapatite structures that support osteogenesis. PLoS One.

[5] Borciani G, Montalbano G, Melo P, Baldini N, Ciapetti G, Vitale-Brovaron C (2021). Assessment of Collagen-Based Nanostructured Biomimetic Systems with a Co-Culture of Human Bone-Derived Cells. Cells.

[6] Ebrahimi Z, Irani S, Ardeshirylajimi A, Seyedjafari E (2022). Enhanced osteogenic differentiation of stem cells by 3D printed PCL scaffolds coated with collagen and hydroxyapatite. Sci Rep.

[7] Kadi F, Dini G, Poursamar SA, Ejeian F (2024). Fabrication and characterization of 3D-printed composite scaffolds of coral-derived hydroxyapatite nanoparticles/polycaprolactone/gelatin carrying doxorubicin for bone tissue engineering. J Mater Sci Mater Med.

[8] Willerth SM, Giles JW, Lindberg G (2023). Editorial: Novel biomaterial strategies for osteogenic treatments. Front Bioeng Biotechnol.

[9] Iaquinta MR, Martini F, D'Agostino A, Trevisiol L, Bersani M, Torreggiani E, Tognon M, Rotondo JC, Mazzoni E (2022). Stem Cell Fate and Immunomodulation Promote Bone Regeneration via Composite Bio-Oss®/AviteneTM Biomaterial. Front Bioeng Biotechnol.

[10] Ruggeri M, Miele D, Caliogna L, Bianchi E, Jepsen JM, Vigani B, Rossi S, Sandri G (2024). Hydroxyapatite-Coated Ti6Al4V ELI Alloy: In Vitro Cell Adhesion. Nanomaterials (Basel).

[11] Patty DJ, Nugraheni AD, Dewi Ana I, Yusuf Y (2022). Mechanical Characteristics and Bioactivity of Nanocomposite Hydroxyapatite/Collagen Coated Titanium for Bone Tissue Engineering. Bioengineering (Basel).

[12] Sun Y, Zhang X, Luo M, Hu W, Zheng L, Huang R, Greven J, Hildebrand F, Yuan F (2021). Plasma Spray vs. Electrochemical Deposition: Toward a Better Osteogenic Effect of Hydroxyapatite Coatings on 3D-Printed Titanium Scaffolds. Front Bioeng Biotechnol.

[13] Wang L, Wang F, Ayisen S, Ren T, Luo X, Wang P (2023). Enhancing the mechanical properties and surface morphology of individualized Ti-mesh fabricated through additive manufacturing for the treatment of alveolar bone defects. Front Bioeng Biotechnol.

[14] Vasile VA, Pirvulescu RA, Iancu RC, Garhöfer G, Schmetterer L, Ghita AM, Ionescu D, Istrate S, Piticescu RM, Cursaru LM, Popa-Cherecheanu A (2024). Titanium Implants Coated with Hydroxyapatite Used in Orbital Wall Reconstruction-A Literature Review. Materials (Basel).

[15] Mysore THM, Patil AY, Hegde C, Sudeept MA, Kumar R, Soudagar MEM (2024). Apatite insights: From synthesis to biomedical applications. Eur Polym J.

[16] Shao H, Zhang Q, Sun M, Wu M, Sun X, Wang Q, Tong S (2023). Effects of hydroxyapatite-coated porous titanium scaffolds functionalized by exosomes on the regeneration and repair of irregular bone. Front Bioeng Biotechnol.

[17] Mazzoni E, Mazziotta C, Iaquinta MR, Lanzillotti C, Fortini F, D'Agostino A, Trevisiol L, Nocini R, Barbanti-Brodano G, Mescola A, Alessandrini A, Tognon M, Martini F (2021). Enhanced Osteogenic Differentiation of Human Bone Marrow-Derived Mesenchymal Stem Cells by a Hybrid Hydroxylapatite/Collagen Scaffold. Front Cell Dev Biol.

[18] Ielo I, Calabrese G, De Luca G, Conoci S (2022). Recent Advances in Hydroxyapatite-Based Biocomposites for Bone Tissue Regeneration in Orthopedics. Int J Mol Sci.

[19] Sagadevan S, Schirhagl R, Rahman MZ, Fareez IM, Lett JA, Fatimah I (2023). Recent advancements in polymer matrix nanocomposites for bone tissue engineering applications. J Drug Deliv Sci Technol.

[20] Nguyen TT, Hu CC, Sakthivel R, Nabilla SC, Huang YW, Yu J, Cheng NC, Kuo YJ, Chung RJ (2022). Preparation of gamma poly-glutamic acid/hydroxyapatite/collagen composite as the 3D-printing scaffold for bone tissue engineering. Biomater Res.

[21] Liu W, Cheong N, He Z, Zhang T (2025). Application of Hydroxyapatite Composites in Bone Tissue Engineering: A Review. J Funct Biomater.

[22] Kavitha Sri A, Arthi C, Neya NR, Hikku GS (2023). Nano-hydroxyapatite/collagen composite as scaffold material for bone regeneration. Biomed Mater.

[23] Hamedi UN, Ciftci F, Soylu TM, Kucak M, Özarslan AC, Altinsoy S (2026). Morphological, Thermal, Mechanical and Cytotoxic Investigation of Hydroxyapatite Reinforced Chitosan/Collagen 3D Bioprinted Dental Grafts. Polymers (Basel).

[24] Yang Y, He H, Miao F, Yu M, Wu X, Liu Y, Fu J, Chen J, Ma L, Chen X, Peng X, You Z, Zhou C (2024). 3D-printed PCL framework assembling ECM-inspired multi-layer mineralized GO-Col-HAp microscaffold for in situ mandibular bone regeneration. J Transl Med.

[25] Veiga A, Madureira S, Costa JB, Castro F, Rocha F, Oliveira AL (2023). Tackling current production of HAp and HAp-driven biomaterials. Mater Adv.

[26] Ciulla MG, Massironi A, Sugni M, Ensign MA, Marzorati S, Forouharshad M (2023). Recent Advances in the Development of Biomimetic Materials. Gels.

[27] Yang P, Ju Y, Hu Y, Xie X, Fang B, Lei L (2023). Emerging 3D bioprinting applications in plastic surgery. Biomater Res.

[28] Hassanin H, Essa K, ElShaer A, Imbaby M, El-Mongy HH, El-Sayed TA (2021). Micro-fabrication of ceramics: Additive manufacturing and conventional technologies. Journal of Advanced Ceramics.

[29] Mollah MdT, Comminal R, Serdeczny MP, Pedersen DB, Spangenberg J (2021). Stability and deformations of deposited layers in material extrusion additive manufacturing. Addit Manuf.

[30] Elhadad AA, Rosa-Sainz A, Cañete R, Peralta E, Begines B, Balbuena M (2023). Applications and multidisciplinary perspective on 3D printing techniques: Recent developments and future trends. Materials Science and Engineering: R: Reports.

[31] Sinha R, Cámara-Torres M, Scopece P, Falzacappa E V, Patelli A, Moroni L (2021). A hybrid additive manufacturing platform to create bulk and surface composition gradients on scaffolds for tissue regeneration. Nat Commun.

[32] Srivastava M, Rathee S, Patel V, Kumar A, Koppad PG (2022). A review of various materials for additive manufacturing: Recent trends and processing issues. Journal of Materials Research and Technology.

[33] Kantaros A (2022). 3D Printing in Regenerative Medicine: Technologies and Resources Utilized. Int J Mol Sci.

[34] Saberi A, Kouhjani M, Mohammadi M, Hosta-Rigau L (2023). Novel scaffold platforms for simultaneous induction osteogenesis and angiogenesis in bone tissue engineering: a cutting-edge approach. J Nanobiotechnology.

[35] Fang L, Lin X, Xu R, Liu L, Zhang Y, Tian F, Li JJ, Xue J (2024). Advances in the Development of Gradient Scaffolds Made of Nano-Micromaterials for Musculoskeletal Tissue Regeneration. Nanomicro Lett.

[36] Maintz M, Tourbier C, de Wild M, Cattin PC, Beyer M, Seiler D (2024). Patient-specific implants made of 3D printed bioresorbable polymers at the point-of-care: material, technology, and scope of surgical application. 3D Print Med.

[37] Larochelle RD, Mann SE, Ifantides C (2021). 3D Printing in Eye Care. Ophthalmol Ther.

[38] Aytac Z, Dubey N, Daghrery A, Ferreira JA, de Souza Araújo IJ, Castilho M, Malda J, Bottino MC (2022). Innovations in Craniofacial Bone and Periodontal Tissue Engineering-From Electrospinning to Converged Biofabrication. Int Mater Rev.

[39] Ivanovski S, Breik O, Carluccio D, Alayan J, Staples R, Vaquette C (2023). 3D printing for bone regeneration: challenges and opportunities for achieving predictability. Periodontol 2000.

[40] Liang W, Zhou C, Zhang H, Bai J, Jiang B, Jiang C, Ming W, Zhang H, Long H, Huang X, Zhao J (2023). Recent advances in 3D printing of biodegradable metals for orthopaedic applications. J Biol Eng.

[41] Gritsch L, Perrin E, Chenal J, Fredholm YC, Maçon ALB, Chevalier J (2021). Combining bioresorbable polyesters and bioactive glasses: Orthopedic applications of composite implants and bone tissue engineering scaffolds. Appl Mater Today.

[42] Qin L, Yang S, Zhao C, Yang J, Li F, Xu Z, Yang Y, Zhou H, Li K, Xiong C, Huang W, Hu N, Hu X (2024). Prospects and challenges for the application of tissue engineering technologies in the treatment of bone infections. Bone Res.

[43] Zhang J, Yarahmadi M, Cabezas L, Serra M, Elizalde S, Cabrera JM (2023). Robocasting of dense 8Y zirconia parts: Rheology, printing, and mechanical properties. J Eur Ceram Soc.

[44] Simorgh S, Alasvand N, Khodadadi M, Ghobadi F, Malekzadeh Kebria M, Brouki Milan P, Kargozar S, Baino F, Mobasheri A, Mozafari M (2022). Additive manufacturing of bioactive glass biomaterials. Methods.

[45] Jongprateep O, Jitanukul N, Saphongxay K, Petchareanmongkol B, Bansiddhi A, Laobuthee A, Lertworasirikul A, Techapiesancharoenkij R (2022). Hydroxyapatite coating on an aluminum/bioplastic scaffold for bone tissue engineering. RSC Adv.

[46] Alkaron W, Almansoori A, Balázsi K, Balázsi C (2024). Hydroxyapatite-Based Natural Biopolymer Composite for Tissue Regeneration. Materials (Basel).

[47] Nitti P, Kunjalukkal Padmanabhan S, Cortazzi S, Stanca E, Siculella L, Licciulli A, Demitri C (2021). Enhancing Bioactivity of Hydroxyapatite Scaffolds Using Fibrous Type I Collagen. Front Bioeng Biotechnol.

[48] Zanca C, Patella B, Capuana E, Lopresti F, Brucato V, Carfì Pavia F, La Carrubba V, Inguanta R (2022). Behavior of Calcium Phosphate-Chitosan-Collagen Composite Coating on AISI 304 for Orthopedic Applications. Polymers (Basel).

[49] Pinto LA, Backes EH, Harb S V, Pinto GM, da Cunha DAL V, Andrade RJE (2023). Shape memory thermoplastic polyurethane/polycaprolactone blend and composite with hydroxyapatite for biomedical application. J Mater Res.

[50] Coffigniez M, Grémillard L, Balvay S, Lachambre J, Adrien J, Boulnat X (2021). Direct-ink writing of strong and biocompatible titanium scaffolds with bimodal interconnected porosity. Addit Manuf.

[51] Zarghami V, Ghorbani M, Bagheri KP, Shokrgozar MA (2022). Improving bactericidal performance of implant composite coatings by synergism between Melittin and tetracycline. J Mater Sci Mater Med.

[52] Szyba D, Kubina R, Młynarek-Żak K, Radoń A, Kania A, Babilas R (2022). Evaluation of the biocompability and corrosion activity of resorbable CaMgZnYbBAu alloys. Sci Rep.

[53] Cardoso GC, de Almeida GS, Corrêa DOG, Zambuzzi WF, Buzalaf MAR, Correa DRN, Grandini CR (2022). Preparation and characterization of novel as-cast Ti-Mo-Nb alloys for biomedical applications. Sci Rep.

[54] Hoppe V, Szymczyk-Ziółkowska P, Rusińska M, Poradowski D, Janeczek M, Ziółkowski G (2021). Study of cytotoxic activity of Ti–13Nb–13Zr medical alloy with different surface finishing techniques. J Mater Sci.

[55] Jhamb SK, Goyal A, Pandey A, Bhowmik A (2025). A review of cytotoxicity testing methods and in vitro study of biodegradable Mg-1%Sn-2%HA composite by elution method. J Mater Sci Mater Med.

[56] Ghezzi D, Boi M, Sassoni E, Valle F, Giusto E, Boanini E, Baldini N, Cappelletti M, Graziani G (2023). Customized biofilm device for antibiofilm and antibacterial screening of newly developed nanostructured silver and zinc coatings. J Biol Eng.

[57] Elmergawy FH, Fathy IA, Abulwafa ZM, Elzayat GA, Seif HM (2025). Fluorinated graphene-modified biodentine: an in vitro study on its ion release, cell growth, differentiation potential, and compressive strength. BMC Oral Health.

[58] Huzum B, Puha B, Necoara RM, Gheorghevici S, Puha G, Filip A, Sirbu PD, Alexa O (2021). Biocompatibility assessment of biomaterials used in orthopedic devices: An overview (Review). Exp Ther Med.

[59] Esposti LD, Marković S, Ignjatović N, Panseri S, Montesi M, Adamiano A (2021). Thermal crystallization of amorphous calcium phosphate combined with citrate and fluoride doping: a novel route to produce hydroxyapatite bioceramics. J Mater Chem B.

[60] Benetti F, de Oliveira PHC, de Andrade MPB, Cantiga-Silva C, Sivieri-Araújo G, Dezan Júnior E, Gomes-Filho JE, Diniz IMA, Dos Reis-Prado AH, Souza MT, Zanotto ED, Cintra LTA (2024). Cytotoxicity, Biocompatibility, and Calcium Deposition Capacity of 45S5 Bioglass Experimental Paste and Bio-C Temp: In Vitro and In Vivo Study Using Wistar Rats. J Funct Biomater.

[61] Omidian S, Haghbin Nazarpak M, Bagher Z, Moztarzadeh F (2022). The effect of vanadium ferrite doping on the bioactivity of mesoporous bioactive glass-ceramics. RSC Adv.

[62] Oladapo BI, Zahedi SA, Ismail SO, Fernando WAM, Ikumapayi OM (2023). 3D-printed biomimetic bone implant polymeric composite scaffolds. The International Journal of Advanced Manufacturing Technology.

[63] Sadek AA, Abd-Elkareem M, Abdelhamid HN, Moustafa S, Hussein K (2023). Repair of critical-sized bone defects in rabbit femurs using graphitic carbon nitride (g-C3N4) and graphene oxide (GO) nanomaterials. Sci Rep.

[64] Yang X, Huang W, Zhan D, Ren D, Ji H, Liu Z, Wang Q, Zhang N, Zhang Z (2022). Biodegradability and Cytocompatibility of 3D-Printed Mg-Ti Interpenetrating Phase Composites. Front Bioeng Biotechnol.

[65] Ou P, Hao C, Liu J, He R, Wang B, Ruan J (2021). Cytocompatibility of Ti-xZr alloys as dental implant materials. J Mater Sci Mater Med.

[66] Depboylu FN, Taşkonak B, Korkusuz P, Yasa E, Ajiteru O, Choi KY (2024). Cleaning and coating procedures determine biological properties of gyroid porous titanium implants. Emergent Mater.

[67] Hadady H, Alam A, Khurana I, Mutreja I, Kumar D, Shankar MR, Dua R (2024). Optimizing alkaline hydrothermal treatment for biomimetic smart metallic orthopedic and dental implants. J Mater Sci Mater Med.

[68] Wang B, Guo Y, Xu J, Zeng F, Ren T, Guo W (2023). Efficacy of bone defect therapy involving various surface treatments of titanium alloy implants: an in vivo and in vitro study. Sci Rep.

[69] Wang Z, Du Y, Zhang S, Li H, Yang J, Yan J, Li Z, Liu J, Liu J (2025). Osteogenic effects of electrophoretically deposited Sr-doped calcium silicate coatings on titanium. Front Bioeng Biotechnol.

[70] Kyyak S, Blatt S, Schiegnitz E, Heimes D, Staedt H, Thiem DGE, Sagheb K, Al-Nawas B, Kämmerer PW (2021). Activation of Human Osteoblasts via Different Bovine Bone Substitute Materials With and Without Injectable Platelet Rich Fibrin in vitro. Front Bioeng Biotechnol.

[71] Kechagias S, Theodoridis K, Broomfield J, Malpartida-Cardenas K, Reid R, Georgiou P, van Arkel RJ, Jeffers JRT (2023). The effect of nodal connectivity and strut density within stochastic titanium scaffolds on osteogenesis. Front Bioeng Biotechnol.

[72] von Heckel C, Walles H, Hasemann G, Krüger M (2025). Modular Ti-6Al-4V system for in vitro optimization of implant materials. Front Bioeng Biotechnol.

